# Cooperative Mechanism of ADAMTS/ ADAMTSL and Fibrillin-1 in the Marfan Syndrome and Acromelic Dysplasias

**DOI:** 10.3389/fgene.2021.734718

**Published:** 2021-11-29

**Authors:** Pauline Arnaud, Zakaria Mougin, Catherine Boileau, Carine Le Goff

**Affiliations:** ^1^ Université de Paris, INSERM U1148, Laboratory for Vascular Translational Science, Hôpital Bichat, Paris, France; ^2^ Département de Génétique, AP-HP, Hôpital Bichat, Paris, France

**Keywords:** ADAMTS, fibrillin-1, Marfan syndrome, acromelic dysplasias, extracellular matrix

## Abstract

The term “fibrillinopathies” gathers various diseases with a wide spectrum of clinical features and severity but all share mutations in the fibrillin genes. The first described fibrillinopathy, Marfan syndrome (MFS), is a multisystem disease with a unique combination of skeletal, thoracic aortic aneurysm (TAA) and ocular features. The numerous *FBN1* mutations identified in MFS are located all along the gene, leading to the same pathogenic mechanism. The geleophysic/acromicric dysplasias (GD/AD), characterized by short stature, short extremities, and joint limitation are described as “the mirror image” of MFS. Previously, in GD/AD patients, we identified heterozygous *FBN1* mutations all affecting TGFβ-binding protein-like domain 5 (TB5). ADAMTS10, ADAMTS17 and, ADAMTSL2 are also involved in the pathogenic mechanism of acromelic dysplasia. More recently, in TAA patients, we identified mutations in *THSD4*, encoding ADAMTSL6, a protein belonging to the ADAMTSL family suggesting that ADAMTSL proteins are also involved in the Marfanoid spectrum. Together with human genetic data and generated knockout mouse models targeting the involved genes, we provide herein an overview of the role of fibrillin-1 in opposite phenotypes. Finally, we will decipher the potential biological cooperation of ADAMTS-fibrillin-1 involved in these opposite phenotypes.

## Introduction

Fibrillins are the major glycoprotein components of microfibrils in the extracellular matrix (Sakai et al., 1986). Through the years, mutations in the fibrillin genes have been identified in various diseases now referred to as fibrillinopathies (Aubart et al., 2016). These diseases represent not only a wide spectrum of disorders including MFS, Marfanoid-progeroid-lipodystrophy syndrome (MFLS), Shprintzen-Goldberg syndrome, acromelic dysplasias such as Weill Marchesani syndrome, GD/AD, but also a wide spectrum of severity, ranging from life-threatening neonatal forms to almost no clinical features in adults. Mutations in *ADAMTS (a disintegrin-like and metalloprotease with thrombospondin type 1 motif)* genes were also identified in acromelic dysplasias and mutations in *THSD4* (encoding ADAMTSL6) in HTAA (heritable thoracic aortic aneurysms) suggesting that the ADAMTS proteins may have a functional link with fibrillin-1. Our review aims to summarize the knowledge focusing on a genetic and biological link between fibrillin-1 and the ADAMTS. We will highlight cooperation between ADAMTS and fibrillin-1 in these human disorders (Table 1).

**TABLE 1:** Genes and disorders associated. This table gathers together all pathologies associated with FBN1 and ADAMTS (-L) proteins mentioned in this review. AD: acromicric dysplasia, ADom: autosomal dominant, AR: autosomal recessive, GD: geleophysic dysplasia, MFS: Marfan syndrome, TAAD: thoracic aortic aneurysm and dissection, WMS: Weill Marchesani syndrome.

Genes	Mendelian disorder associated	Mode of inheritance	Main clinical features	References
***FBN1***	MFS/TAAD	ADom/AR	TAAD, ectopia lentis, tall stature	Arnaud et al. (2017), Arnaud et al. (2019)
	WMS	ADom	Short stature, brachydactyly, restricted joint mobility, thick skin, microspherophakia, ectopia lentis	Faivre, (2003)
	GD	ADom	Short stature, brachydactyly, restricted joint mobility, thick skin, valve stenosis that may lead to death	Le Goff et al. (2011)
	AD	ADom	Short stature, brachydactyly, restricted joint mobility, thick skin, hoarse voice, internal notch of the femoral head	Le Goff et al. (2011)
**LTBP**				
*LTBP2*	WMS-like	AR	Short stature, brachydactyly, ectopia lentis	Haji-Seyed-Javadi et al. (2012)
*LTBP3*	AD	ADom	Short stature, brachydactyly, restricted joint mobility, thick skin, hoarse voice, internal notch of the femoral head	McInerney-Leo et al. (2016)
	GD	ADom	Short stature, brachydactyly, restricted joint mobility, thick skin, valve stenosis that may lead to death, severe respiratory disease	McInerney-Leo et al. (2016)
	TAAD	ADom/AR	TAAD with skeletal and dental phenotypes	Guo et al. (2018)
**ADAMTS(L)**				
*ADAMTS10*	WMS	AR	Short stature, brachydactyly, restricted joint mobility, thick skin, microspherophakia, ectopia lentis	Dagoneau et al. (2004), Kutz et al. (2008)
*ADAMTS17*	WMS-like	AR	Short stature, microspherophakia, ectopia lentis	Morales et al. (2009)
*ADAMTSL2*	GD	AR	Short stature, brachydactyly, restricted joint mobility, thick skin, cardiac valvular abnormalities, tip-toe walking	Le Goff et al. (2008), Allali et al. (2011)
*THSD4* (ADAMTSL6)	TAAD/Marfanoid	ADom	MFS-like (TAAD)	Elbitar et al. (2021)
**SMAD**				
*SMAD3*	TAAD/Marfanoid	ADom	TAAD and AOS (aneurysm-osteoarthritis syndrome)	van de Laar et al. (2011)
*SMAD4*	Myhre Syndrome	ADom	Short stature, brachydactyly, cardiac manifestations, deafness, intellectual disabilities	Le Goff et al. (2012)
	TAAD	ADom	TAAD	Duan et al. (2019)

## Marfan Syndrome and Related Conditions

### Marfan Syndrome

#### Clinical Features

MFS (OMIM #154700) is the best-known fibrillinopathy. It is a rare connective tissue disorder, with an estimated prevalence of around 1 out of 5,000 individuals, which affects different systems, including the cardiovascular system, but also the skeleton, the eye, and sometimes the skin, the lung, and the neurological system. The clinical variability is high, either in the age of appearance of the symptoms or in their number or severity (Arnaud et al., 2019).

##### Cardiovascular Manifestations

Cardiovascular manifestations with TAA or dissections are the most serious life-threatening complications of this syndrome. TAA is characterized by a dilatation, which is predominant at the root of the aorta called the sinus of Valsalva. Its evolution is nonlinear and thus it is not possible to predict the dilatation rate or the risk of dissection (Detaint et al., 2010). Men appear to be at higher risk for an aortic event than women (Détaint et al., 2010). Hypertension, bicuspid aortic valve, and pregnancy are factors that can increase the risk of dilatation and dissection (Renard et al., 2017). There is no curative treatment, but three methods are currently used to control this cardiovascular risk: controlled lifestyle and sport restrictions, life-long β-blocker therapy, and preventive aortic root replacement surgery (Erbel et al., 2014). Indications for surgery are based mainly on aortic diameter and derived from findings on familial history regarding the risk of complications. Surgery should be performed in patients with MFS, who have a maximal aortic diameter ≥50 mm compared to 55 mm in non-genetic TAA (Jondeau et al., 2012). Another important cardiovascular feature is mitral valve prolapses and it can be complicated by mitral valve insufficiency.

##### Ocular and Skeletal Manifestations

The eye is another predominant system involved in MFS. Ectopia lentis, which is due to hypermobility of the ciliary zonule, is a typical feature in MFS, as well as myopia and flat cornea. The disease can lead to lens dislocation or retinal detachment and possibly blindness. The treatment of ectopia lentis is surgical often in childhood (Arnaud et al., 2021a).

The skeleton and ligaments can also be altered in MFS with the occurrence of arachnodactyly with positive wrist and/or thumb signs, dolichostenomelia, anterior chest deformity, scoliosis, tall stature, hypermobility, and pes planus. Another connective tissue, the skin can also be altered with striae and herniae, as well as the lung and the dura matter with spontaneous pneumothorax and dural ectasia (lumbosacral meningocele), respectively.

#### Diagnosis of Marfan Syndrome

The different clinical features of MFS require the expertise of physicians from different medical specialties: cardiologists, ophthalmologists, rheumatologists or pediatricians (depending on the age), and geneticists. Systematic slit-lamp examination, cardiac ultrasonography, and radiological investigations should also be performed. Dural ectasia should be looked for by imaging.

The diagnosis of MFS is established according to the revised Ghent nosology (Loeys et al., 2010), which is recognized by international experts. The Ghent criteria comprise a set of major and minor manifestations in different body systems. A systemic score can be established to assess the importance of systemic features of MFS. This score, along with the status of the thoracic aorta, the presence of ectopia lentis, and *FBN1* genetic testing, are the basis of the Ghent nosology.

#### Molecular Basis

The mode of inheritance in MFS families is usually autosomal dominant. Family history is documented in approximately 65% of MFS cases (Arnaud et al., 2021b), while the remaining are sporadic cases. Patients with MFS usually carry heterozygous mutations in the *FBN1* gene, encoding for fibrillin-1, an extracellular matrix (ECM) protein (Collod-Béroud et al., 2003). All types of mutations are described in the *FBN1* gene, ie loss-of-function mutations (splicing mutations, exon(s) deletion/duplication or insertion/deletion, and missense mutations). The numerous *FBN1* pathogenic variants identified in MFS are located throughout the gene, with no specific clustering except for neonatal and severe forms of MFS associated with mutations within the exons 24 to 32 (called the “neonatal” region). Some cases of symptomatic MFS probands harboring a mosaic *FBN1* pathogenic variant have been recently described (Arnaud et al., 2021b). Finally, rare instances of apparent autosomal recessive transmission of MFS were found to be associated with hypomorphic variants in homozygous subjects (Arnaud et al., 2017). Patients with homozygous or compound heterozygous variants present either a classical form of MFS or a severe form of clinical presentations suggesting that these mutations are not associated with a prediction of the severity of the disease.

Two pathogenic mechanisms have been associated with *FBN1* mutations in MFS: negative dominance (since microfibrils are fibrillin polymers) and haploinsufficiency. The largest report for haploinsufficiency was an *FBN1* expression study in skin fibroblasts from 80 MFS patients harboring premature termination codon (PTC) variants (Aubart et al., 2015). Under this model, a correlation between *FBN1* expression in skin fibroblasts and some MFS features, especially ectopia lentis and pectus deformity, was demonstrated.

An important part of missense mutations found in MFS patients affects the cysteine residues of fibrillin-1 (Collod-Béroud et al., 2003).

The mutations located within the neonatal region (corresponding to exons 24–32) have been associated with more severe phenotypes, including neonatal MFS (Faivre et al., 2007). Neonatal MFS is a very early-onset form of the disease that is diagnosed between birth and the third month of life (Stheneur et al., 2011). These young patients display severe cardiovascular features (TAA and mitral and valvular dysfunction), pulmonary emphysema, joint contractures, crumpled ears, and cutis laxa. Some cases with more classical symptoms such as arachnodactyly and ectopia lentis are also found (Hennekam, 2005).

It has been recently shown that *FBN1* genotype-phenotype correlations exist for both aortic and extra-aortic features (Arnaud et al., 2021a). In this study including probands and relatives carrying a mutation in the *FBN1* gene, PTC variants were not only associated with a shorter life expectancy and a high lifelong risk of an aortic event but also with the highest risk of severe scoliosis as well as a lower risk for ectopia lentis surgery. In-frame variants could be subdivided according to their impact on the cysteine content of fibrillin-1 with a global higher severity for cysteine-loss variants and the highest frequency of EL surgery for cysteine-addition variants. These correlations suggest different pathological mechanisms in MFS depending on the impact at the protein level in various organs.

### Related Conditions of Marfan Syndrome

Related conditions to MFS are all the disorders with similar clinical features as MFS but with no causal mutations in the *FBN1* gene. Recently, mutations in *THSD4*, encoding ADAMTSL6, and in *LTBP3* have been described in this MFS spectrum.

#### Condition Linked to *THSD4*

##### Clinical Features

Patients with mutations in *THSD4* present a Marfanoid phenotype, with aortic events (aortic dilation, aortic dissection), and skeletal features such as mild pectus excavatum, scoliosis but with no ocular manifestations.

##### Molecular Basis

Using whole-exome sequencing on a cohort of heritable TAA and marfanoid patients without a known molecular basis, we identified a total of five pathogenic heterozygous variants in the *THSD4* gene that encodes the extracellular protein ADAMTSL6. These variants consist of two PTC mutations, one 9bp in-frame duplication, and two missense mutations, all leading to loss of function through haploinsufficiency or abnormal network of fibrillin-1 microfibrils (Elbitar et al., 2021).

#### Condition Linked to *LTBP3* Gene

##### Clinical Features

Patients carrying *LTBP3 (Latent TGFβ-binding protein 3)* variants present aneurysms and dissections of the thoracic aorta, as well as aneurysms of the abdominal aorta and other arteries. They display also dental abnormalities and short stature. Heterozygous carriers of the *LTBP3* variant have a later onset of thoracic aortic disease, associated with dental abnormalities. Interestingly, heterozygous rare LTBP3 variants were also identified in cases with early onset of acute aortic dissections (Guo et al., 2018).

##### Molecular Basis

Using whole-exome sequencing on a cohort of heritable TAA and dissection without a known molecular basis, *LTBP3* has been identified as a good candidate gene (Guo et al., 2018). These variants were homozygotes and compound heterozygotes impacting mostly the calcium-binding epidermal growth factor (cbEGF) repeats of the protein LTBP3 as well as LTBP3 protein level. *Ltbp3* ^*-/-*^ mouse model presented short stature compared to wild-type and heterozygotes, with reduced diastolic blood pressure, and increased ascending aortic diameter associated with dysregulated elastic fibers. This phenotype of this mouse model validated the potential involvement of LTBP3 in HTAA.

## Acromelic Dysplasias

Acromelic dysplasias are a group of twelve rare skeletal disorders impacting the connective tissue, this review will focus on three of these disorders namely: Weill-Marchesani Syndrome (WMS), GD, and AD. All are characterized by short stature, shortened extremities, thick skin, and limited joint mobility, but can be distinguished by specific genetic and clinical features described below. This review focuses on acromelic dysplasias associated with *FBN1* and *ADAMTS(-L)* mutations.

### Weill-Marchesani Syndrome

#### Clinical Features

First described in 1932 by Weill (Weill, 1932), a woman presented ectopia lentis, short stature, and stubby, swollen fingers. In 1939, Marchesani (Marchesani, 1939) described two unrelated families presenting short stature and short extremities. Since parental consanguinity was demonstrated in one of the families, Marchesani suggested the pathology to be a “recessive condition”. The incidence of WMS in different populations is one over 100,000 births (Marzin et al., 1993). WMS (OMIM#608328) is characterized by short stature, brachydactyly, limited joint mobility, stubby hands, and feet, stiff joints, and thickened skin, especially in the hands. Most patients have been described with ophthalmic features, among them dislocation of microspherophakic lenses which causes myopia, ectopia lentis (Mcgavic, 1966), acute and/or chronic glaucoma (Lim et al., 2016), and cataract. Cardiac defects in WMS include aortic and pulmonary valve stenosis, mitral stenosis (Vandewoestijne, 2004), valvular thickening, mitral valve prolapse, corrected QT interval (QTc) prolongation on electrocardiogram (ECG) (Kojuri et al., 2007), and more recently cervical artery dissection (Newell et al., 2017) and acute thoracic dissection (Cecchi et al., 2013).

#### Molecular Basis

In 1996, with linkage analysis of two new unrelated families, Wirtz et al. suggested that the WMS locus was on chromosome 15q21.1, where the *FBN1* gene maps. The disease was associated with the decrease of FBN1 protein in the dermal-epidermal junction (Wirtz et al., 1996). In 2003, Faivre et al. first identified heterozygosity for a 24-bp in-frame deletion in exon 41 of *FBN1* which segregated with the disease. This deletion causes a loss of eight residues in one of the 7 TB domains (Faivre, 2003). Since then, several mutations in *FBN1* have been described leading to WMS, clustered in exons 41 and 42 (Cecchi et al., 2013).

Mutations in *ADAMTS10* (OMIM#277600) and *ADAMTS17* genes (OMIM#613195) are responsible for the autosomal recessive form of WMS. In 2002, Faivre et al. first described by homozygosity mapping in consanguineous WMS Lebanese and Saudi families a locus on chromosome 19p13.3-p13.221 that led to the identification of homozygous nonsense and splicing mutations in *ADAMTS10* (Faivre et al., 2002; Dagoneau et al., 2004). Mutations were located in exons 6 and 10 encodings the metalloprotease domain of ADAMTS10. Compound heterozygous p. (Ala25Thr) and p. (Asp318*) mutations in *ADAMTS10* were also identified in a sporadic case of WMS (Kutz et al., 2008).

In 2009, Morales et al. described 8 individuals from two Saudi Arabian families and two sporadic cases with a “Weill-Marchesani-like” phenotype due to the absence of brachydactyly and limited joint mobility. Linkage analyses and targeted sequencing led to the identification of three homozygous mutations in the *ADAMTS17* gene (Morales et al., 2009). In another consanguineous Saudi Arabian family described in 2012, a new homozygous frameshift mutation in the *ADAMTS17* gene led to short stature and spherophakia (Khan et al., 2012).

In a consanguineous Indian family, a woman was described with microspherophakia, short stature, and brachydactyly, but again no limited joint mobility was observed. Performing whole-exome sequencing for this woman and unaffected relatives, a homozygous splice mutation in the *ADAMTS17* gene was identified (Shah et al., 2014).

Thus, both autosomal dominant (ADom) and recessive (AR) modes of inheritance have been described for WMS. All cases shared short stature, brachydactyly, thick skin, ocular defects, thus illustrating some clinical homogeneity. However, depending on the mode of inheritance, significant results showed that some clinical features are more likely to appear in some patients. For instance, microspherophakia was found in 94% of AR patients harboring *ADAMTS10* mutations but only in 74% of ADom patients, carriers of *FBN1* mutations, or joint limitations being found in 49% of AR cases and 77% of ADom cases. The authors conclude that WMS presented clinical homogeneity with genetic heterogeneity (Faivre et al., 2003).

In 2012, Haji-Seyed-Javadi screened thirty unrelated Iranian probands with families with ectopia lentis which four were associated with WMS. Due to the absence of disease-causing mutations in already-known genes (*e.g*., *FBN1*, *ADAMTS10,* and *ADAMTS17*) and the consanguineous parents in some families, they performed a whole-genome homozygosity mapping and found *LTBP2* (Haji-Seyed-Javadi et al., 2012) to be the only homozygous region segregating with the affected status. Compound heterozygous *LTBP2* mutations can also cause the disease (Lin et al., 2021, 2).

### Geleophysic Dysplasia

#### Clinical Features

First described in 1971, Spranger et al. presented two patients with short stature, short hands, and feet, limited joint mobility, brachydactyly, ovoid vertebrae, and hepatomegaly (Spranger et al., 1971). They hypothesized that the patient described by Vanace et al., in 1960, who died of cardiac defect, could also have the same disorder (Vanace et al., 1960). They suggested the term “geleophysic” due to the happy appearance of patients’ faces (from Greek *gelios* “happy”, *physis* “nature”). The prevalence of GD is estimated at less than one over 1,000 000 births (Cheng et al., 2018). GD is characterized by the main phenotype features of acromelic dysplasias such as short stature, brachydactyly, limited joint mobility, thickened skin. The cardinal distinctive characteristics are facial features: a “happy” face with full cheeks, thin upper lip, long flat philtrum, shortened nose, and hypertelorism. GD patients also present progressive cardiac valvular thickening generally leading to an anticipative death before 5 years of age. They can develop severe respiratory insufficiency due to bronchial and tracheal stenosis which can also lead to the early death of these patients (Le Goff and Cormier-Daire, 2009). Skeletal manifestations include delayed bone age, cone-shaped epiphyses, shortened long tubular bones, and ovoid vertebral bodies. Improper contractions of the Achilles tendon and gastrocnemius muscle lead to tiptoe walking (Le Goff et al., 2008). GD is the most severe acromelic dysplasia.

#### Molecular Basis

In 2008, using homozygosity mapping in four unrelated consanguineous families and two unrelated non-consanguineous families, the locus segregating with GD was identified on chromosome 9q34.2 and shown to be the *ADAMTSL2* gene (Le Goff et al., 2008). Mutations were found either in the cysteine-rich module or in the sixth thrombospondin type I repeat (TSR6). Following this initial study, 33 other patients were screened for *ADAMTSL2* mutations (OMIM#231050). In this second analysis, mutations were identified all along with the protein (Allali et al., 2011). To date, 29 *ADAMTSL2* mutations (18 missense, 4 nonsense, 2 deletions, and 5 affecting splicing) have been reported in 43 families with GD without a clear genotype/phenotype correlation (Piccolo et al., 2019). Genetic heterogeneity in GD was demonstrated (Allali et al., 2011). In 2011, a total of fifteen mutations (14 missenses and one insertion) in the *FBN1* gene (OMIM#614185), were identified using whole-exome sequencing (Le Goff et al., 2011). All were clustered in the TB5 domain of FBN1. The involvement of a cysteine residue in an *FBN1* missense variant is statistically associated with heart valve defects (Marzin et al., 2021).

Due to the absence of a molecular basis for some GD patients, the conclusion was made that *ADAMTSL2* and *FBN1* are not the only genes involved in the development of the disorder. Whole-exome sequencing was used to identify heterozygotes mutations in the *LTBP3* gene, especially a loss of in-frame STOP codon (McInerney-Leo et al., 2016).

### Acromicric Dysplasia

#### Clinical Features

First described in 1986 by Maroteaux et al., six sporadic patients presenting short stature, mild facial anomalies, and shortened hands and feet (Maroteaux et al., 1986), AD (OMIM#102370) is characterized by short stature gradually acquired postnatally, short hands and feet, joint limitations, and skin thickening (Maroteaux et al., 1986; Faivre et al., 2001). The prevalence of AD in different populations is one over 1,000 000 births AD clinical features differ from those of GD with recognizable facial features (e.g., round face, bulbous nose with anteverted nostrils, long and prominent philtrum, thick lips with small mouth), skeleton features (e.g., an internal notch of the femoral head, internal notch of the second metacarpal, and external notch of the fifth metacarpal), a hoarse voice and a pseudomuscular build. No cardiac defects have been observed.

#### Molecular Basis

Vertical transmission of AD in three families and 16 sporadic cases described by Faivre et al. (2001) demonstrated the autosomal dominant mode of inheritance of the disorder. In 2011, Le Goff et al. identified, in ten AD patients, including two families, eight heterozygous missense mutations, and one heterozygous duplication (Le Goff et al., 2011). As in GD, all were clustered in exons 41 and 42 of the *FBN1* gene, encoding TB5. Interestingly, some mutations were shared with GD. To date, in the literature, a total of 23 mutations are described in the *FBN1* gene in patients with GD or AD (Le Goff et al., 2011; Lee et al., 2013; Wang et al., 2014, 2020; Jin et al., 2017; Globa et al., 2018; Sun et al., 2020).

Heterozygote missense mutations in *LTBP3* also lead to AD. All mutations cluster in the calcium-binding EGF domain of the protein LTBP3 (McInerney-Leo et al., 2016).

## Fibrillin-1

### FBN1 Microfibrils Assemblage and Involvement in the Extracellular Matrix Biogenesis

In 1986, Sakai et al. report for the first time the existence of fibrillin 1 (FBN1) as long flexible molecules that are major structural components of 12–20 nm diameter microfibrils (Sakai et al., 1986). This key structural function was largely reported during the first decades of the identification and description of fibrillins. (Ramirez and Sakai, 2010). Fibrillin-1 is a multidomain glycoprotein of 350 kDa, comprising 47 epidermal growth factor-like domains, among which 43 are calcium-binding (cbEGF-like), 7 transforming growth factor-β (TGF-β)-binding protein domains (TB), 2 hybrid domains, a proline-rich domain, and the N- and C-terminal domains. Fibrillins are the extracellular proteins with the highest content of cysteine residues (14%) (Sakai et al., 1986). Indeed, EGF-like domains contain 6 cysteine residues while TB and hybrid domains contain 8 to 9. Cysteine residues are implicated in disulfide bonds which are known to play a major role in fibrillin structure and function. An RGD integrin-binding motif is localized in the TB4 domain. And four distinct heparin-binding sites are contained in fibrillin-1 (Ramirez and Sakai, 2010). Once secreted, fibrillin protein monomers constitute microfibrils in the ECM of connective tissues. The function and the structure of microfibrils depend on the tissue type. In the aortic wall, for example, microfibrils are the scaffold for the deposition of tropoelastin, a soluble precursor that coacervates into elastin chains and forms mature elastic fibers. In 2004, Mizuguchi and al described for the first time several mutations in *TGFBR2* (encoding the TGF-β receptor, type 2) in MFS patients (Mizuguchi et al., 2004). This led to considering the microfibril scaffold as a reservoir of growth factors (in particular TGF-β) (Sengle and Sakai, 2015). However, these two roles of microfibrils, ie structural and signaling roles, differ between the stage of life, the tissue, and the type of fibrillin.

*FBN1* is ubiquitously expressed, even during development, with an expression ranging from high levels in skin fibroblasts to quite low in the central nervous system (Quondamatteo et al., 2002; Hubmacher et al., 2006).

FBN1 proteins are assembled either in a head-to-tail arrangement and laterally at the cell surface (Charbonneau et al., 2010). However, the molecular basis of the assembly of these highly complex polymers is not completely understood. It has been shown that a central FBN1 sequence binds with high-affinity and crosslinks with tropoelastin which confirms that their association is fundamental to elastic fiber formation (Kielty et al., 2005). It allows the adaptation of elastic fibers to different tissue-specific organizations with different mechanical requirements. For example, in the arterial wall, in which fibrillins are synthesized by vascular smooth muscle cells (vSMCs) during development, elastic fibers must be organized as fenestrated concentric rings that support thoracic aorta compliance. Afterwards, the phenotype of vSMC change from a role in biosynthesis to contraction and maturation of the ECM. Thus, in the thoracic aorta, the mechanical properties of elastin fibers and vSMCs support the elasticity. On the contrary, in the dermis, microfibrils and elastic fibers form a loose meshwork which contribute to skin pliability (Ramirez and Sakai, 2010).

Microfibrils are associated with other proteins in the ECM of connective tissues. Multiple proteins are known to bind closed to the N-terminus of fibrillin-1 such as LTBPs, fibulin-1, -5, versican, and microfibril-associated proteins 1 (MAGP1) (Gibson et al., 1996; Ono et al., 2009; Thomson et al., 2019). This similar location of their binding sites suggests that depending on the tissue, each associated protein may give to fibrillin-1 a different role. Indeed, depending on the tissue (*e.g.,* eye or skin), the FBN1 protein does not exhibit the same primary structure (bead morphology and periodicity). The composition of the microfibrils presented also certain variations (more elastic fiber associated proteins). This tends to correlate the different roles of FBN1 to distinct tissues where they are present (Eckersley et al., 2018).

### FBN1 Microfibrils and Cytokines

Fibrillins, as ECM proteins involved in the storage and regulation of growth factors, act as a reservoir of cytokines in which they are sequestratedSome of these interactions have been described as playing a major role in the pathophysiology of TAA such as MAGP2 (also known as MFAP5) and TGF-β (Regalado and Milewicz, 2015). Indeed, mutations in the genes encoding these proteins and in genes encoding other downstream actors of this signaling pathway [*TGFB2* (Boileau et al., 2012), *MFAP5* (Barbier et al., 2014) *TGFBR1* (Loeys et al., 2005); *TGFBR2* (Mizuguchi et al., 2004) and *SMAD3* (van de Laar et al., 2011)] are involved in familial TAA.

TGF-β cytokines are synthesized as protein precursors. The propeptide of TGFβ, the latency-associated peptide (LAP) shelters TGFβ. Furin convertase cleaves the pro dimer of TGFβ to produce the small latent TGFβ complex (SLC). The SLC can covalently bind to the large latent TGFβ binding proteins (LTBPs) to form the large latent complex (LLC) (Saharinen and Keski-Oja, 2000). LTBPs (LTBPs 1–4) are members of the fibrillin family of proteins. They have a similar modular organization to fibrillins including repeating cysteine-rich domains, epidermal growth factor (EGF)-like repeats, eight cysteine domains, or transforming growth factor protein-binding-like domains (also called TB domains). The C-terminus of LTBP1 binds to the N-terminal region of fibrillin-1 leading to a link between LLC and elastic fibers (Isogai et al., 2003). LTBP also interacts with other ECM components such as fibronectin. In this way, microfibrils can be considered as a regulator of a cytokine such as TGFβ.

Various mechanisms can occur to activate TGFβ. As part of TGFβ activation, fibrillin fragments are produced after proteolysis by elastases and particularly an internal fragment that has an affinity with the N-terminal region of fibrilllin1. This interaction between this fragment of fibrillin and fibrillin microfibrils leads to the displacement of LTBP and the release of the LLC, thus contributing to the activation of TGFβ (Chaudhry et al., 2007). BMP1 (a metalloendopeptidase) also has been shown to have a role in the release of LLC by cleaving LTBP1 at two specific sites in the hinge region to release LLC. The integrins α_v_β_6_ and α_v_β_8_ are involved in the enhancement of TGF-β activation through the force-mediated liberation (Wipff and Hinz, 2008; Aluwihare et al., 2009).

Once the active and mature TGFβ is released in the ECM, binding to cellular receptors may occur. TGFβ binds to the TGFβ type II receptor (TGFBR2) that recruits and phosphorylates TGFBR1 (also known as ALK5) in the vast majority of cell types. TGFBR1 then recruits and phosphorylates a receptor-regulated SMAD protein (R-SMAD), SMAD2, and/or SMAD3. These SMAD proteins form a complex with the common-mediator SMAD4 (co-SMAD) to transduce extracellular signals to the nucleus to activate targeted gene transcription (ten Dijke and Arthur, 2007). Many genes are regulated by SMAD signaling, especially those encoding ECM components and regulators (collagens, metalloproteases MMPs and their tissue-inhibitors TIMPs, etc.). TGF-β receptors also activate other signaling pathways, as the ERK and JNK cascade. It is now widely accepted that along with structural defects, MFS and TAA pathophysiology also includes excess active TGF-β signaling and dysregulation between the SMAD and ERK, and JNK pathways (Regalado and Milewicz, 2015).

In opposite to TGFβ, it is important to notice that fibrillin-1 can bind directly to bone morphogenetic proteins (BMPs) such as BMP2, 4, and 10 (Sengle et al., 2012).

## ADAMTS and ADAMTSL

ADAMTS (A Disintegrin like And metalloproteinase with ThromboSpondin type 1 motif) includes a family of 19 secreted enzymes, with its first member -ADAMTS1- being discovered in 1997 (Kuno et al., 1997). All ADAMTS share the same domain organization: at the N-terminus a catalytic domain, and the C-terminus an ancillary domain crucial for distinct interaction with their substrates. In opposite to the membrane attached ADAMs, ADAMTS contain one or several thrombospondin type I repeats (TSR) (Apte, 2009). They are synthesized as inactive zymogens, activated through furin cleavage of their N-terminal propeptide. Each ADAMTS belongs to an evolutionary group according to their homology between ADAMTS and their specific substrates, for example, the procollagen N-propeptidase group gathers ADAMTS2, 3, and 14.

In diverse physiological biological processes as well as pathological, the ADAMTS family has been associated with, including cancer, angiogenesis, cell migration, connective tissue structure. Through ECM degradation and turnover, these matrix metalloproteases are also involved in the proteolysis of cell surface and soluble proteins. (Le Goff and Cormier-Daire, 2011; Dubail and Apte, 2015). Interestingly, the C-terminal of certain ADAMTS can be proteolyzed leading to the production of ADAMTS fragments that have specific functions (Kashiwagi et al., 2004; Li et al., 2009).

The ADAMTS-like (ADAMTSL) proteins are similar to the ADAMTS ancillary domain and by consequence do not possess any enzyme activity (Dubail and Apte, 2015). They are not issued from alternative splicing of ADAMTS genes. At least seven ADAMTSL proteins belong to this group, and their function is still unknown. A potential role of inhibitor or enhancer of ADAMTSL on ADAMTS has been suggested. A *Drosophila* ADAMTSL named papilin was demonstrated to be a non-competitive inhibitor of ADAMTS2 *in vitro* using purified proteins (Kramerova et al., 2000).

ADAMTS and ADAMTSL protein are also involved in the microfibrils and matrix stability (Kutz et al., 2011; Le Goff et al., 2011; Sengle et al., 2012) but also in cell adhesion (Cain et al., 2016).

## The Biological Link Between ADAMTS/ADAMTSL Proteins and FBN1

### Interaction Between ADAMTS and FBN1

Due to the emergence of mutations in ADAMTS and ADAMTSL proteins in the disorders’ spectrum, phenocopying the fibrillinopathies, more questioning has been made about the roles of these proteins in the microfibrillar network biogenesis and homeostasis, and more specifically their interactions with FBN1. Mostly using surface plasma resonance, the interaction between FBN1 and ADAMTS and ADAMTSL proteins has been investigated (Table 2). First ADAMTS10 colocalizes with FBN1 microfibrils in the skin and the ciliary zonule. ADAMTS10 directly interacts with FBN1 (Kutz et al., 2011), specifically at the N-terminus extremity of the protein between the second EGF repeat and the first cbEGF repeat (Cain et al., 2016), but also in the C-terminus half between the TGF-β binding domain 6 and 7 (Kutz et al., 2011). ADAMTSL2 directly interacts with FBN1 through the N-terminus extremity (Le Goff et al., 2011) and more precisely between the TGF-β binding domain 1 and the last EGF repeat for ADAMTSL2 (Sengle et al., 2012). ADAMTSL6 binds to fibrillin-1 more precisely at its half N-terminus (Tsutsui et al., 2010). Interestingly, in cell culture, ADAMTS17 is not able to bind to fibrillin-1 but colocalizes with microfibrils. All these ADAMTS have similar binding sites on fibrillin-1 raising the potentiality that a very precise regulation should exist between all these ADAMTS depending on the tissues and the stage of development.

**TABLE 2:** *In vitro* and *in vivo* fibrillins partners.

	Partners	Function	Interaction showed	References
**FBN1**	ADAMTS10	Microfibrils biogenesis and homeostasis	*In vitro/in vivo*	Kutz et al. (2011); Mularczyk et al. (2018)
	ADAMTSL2	Microfibrils assembly and composition	*In vitro*	Le Goff et al. (2008); Delhon et al. (2019); Hubmacher et al. (2019)
	ADAMTSL6	Microfibrils biogenesis	*In vitro*	Tsutsui et al. (2010)
	LTBP2	Microfibrils biogenesis	*In vivo/in vitro*	Fujikawa et al. (2017)
	LTBP3	LTBP3 incorporation into the microfibrillar network	*In vitro*	Zilberberg et al. (2012)
	FBN2	Microfibrillar network	*In vitro*	Lin et al. (2002)
**ADAMTS17**	FBN2	Microfibrils biogenesis	*In vitro*	Hubmacher et al. (2017)

### FBN1 in Marfan Syndrome and Thoracic Aortic Aneurysm and Dissection

The pathomechanism of MFS seems to be a combination of a loss of mechanical function of fibrillin 1-rich microfibrils and an impairment of the TGFβ signaling pathway. Characterization of mice with ubiquitous or cell type-restricted fibrillin-1 deficiency has unraveled some pathophysiological mechanisms associated with the MFS phenotype, such as altered mechanotransduction in the heart, dysregulated TGF-β signaling in the ascending aorta, and perturbed stem cell fate in the bone marrow (Smaldone et al., 2016).

To elucidate the mechanism implicated in the onset of MFS, two transgenic mouse models, *Fbn1* ^C1663R^ and *Fbn1* ^C1039G^, both involve a cysteine residue, which represents an important part of missense mutations found in MFS patients, were generated (Judge et al., 2004). In humans, these two mutations (p.Cys1039Tyr and p. Cys1663Arg) were found in classical forms of MFS. *Fbn1* ^C1663R^ mice, showed no abnormality, while *Fbn1* ^C1039G^ heterozygous knock-in mice (*Fbn1* ^C1039G/+^) displayed a phenotype similar to MFS patients. Indeed, *Fbn1* ^C1039G/+^ mice had a normal life span and did not die because of aorta dissection, but after 2 months of life, a progressive deterioration of the aortic media was observed including elastic fiber fragmentation, diminished FBN1 network, and thickened aortic wall. Moreover, these mice presented skeletal abnormalities, kyphosis, and rib overgrowth. Finally, the introduction of a wild-type human *FBN1* transgene rescued the aortic phenotype. These observations suggested that *FBN1* haploinsufficiency is the primary determinant of failed microfibril assembly.

A few years later, to gain further insight into physiopathology and the effect of mutant *FBN1* on the assembly and stability of microfibrils, Charbonneau et al. (2010) generated two knock-in mouse lines, GT-8 and H1Δ. H1Δ mice, which have an in-frame deletion of the first hydride domain (H1) in the *Fbn1* gene, showed no abnormal phenotype or alteration of microfibril structure and function. This domain was chosen since it is involved in LTBPs and FBN1 interactions (Ono et al., 2009). In the GT-8 mouse model, the *Fbn1* gene was truncated (from exon 32 until the end) and tagged with an enhanced green fluorescent protein (eGFP). Homozygous mice were not viable, whereas, in heterozygous mice, fragmentation of elastic lamellae in the aorta was observed. In these mice, a truncated FBN1 was secreted like the wild-type protein and assembled into microfibrils. Therefore, truncated FBN1 did not interfere with microfibril assembly but exerted a dominant-negative effect on the stability of microfibrils.

### FBN1 and ADAMTSL6

The recent identification of *THSD4* as a new gene involved in TAA led us to consider this ADAMTSL protein even seem implicated in the Marfanoid spectrum. Indeed, certain patients carrying *THSD4* mutations presented skeletal defects but no ocular anomalies (Elbitar et al., 2021). *THSD4* encodes the ADAMTSL6 protein which is known to be an extracellular protein involved in microfibrillar biogenesis. Using whole mouse embryos and adult tissues, its tissue expression pattern was evaluated by immunohistochemistry (Tsutsui et al., 2010). In E16.5 mouse embryos, ADAMTSL6 proteins were detected in fibrillar structures in various elastic tissues, such as the dermis, perichondrium, and the aortic wall. Interestingly, ADAMTSL6 was also present, in adult kidney artery walls and the mitral valve in the adult heart. All these data suggest that ADAMTSL6 is an ECM protein associated with elastic tissues in their fibrillary components. Our recent data supported that *Thsd4* ^+/-^ mice present progressive thoracic aortic dilation with disruption of elastic fibers and increased apoptosis of SMCs. ADAMTSL6 interacts directly with fibrillin-1 and promotes early-stage fibrillin-1-microfibril assembly (Tsutsui et al., 2010), and indirectly regulates the TGFβ signaling pathway in the cancer context (Liu et al., 2021).

In transfected HEK293 cells, the presence of mutations in *THSD4* leads to impairments of FBN1 microfibril assembly. In the aorta of the mutated patients, the disorganization of the FBN1 microfibril network was confirmed and was associated with an increase in the protein rate of TGFβ and overactivation of the TGFβ signaling pathway (Elbitar et al., 2021). Through these results, the role of ADAMTSL6 in the homeostasis of FBN1 in the aorta was highlighted.

### FBN1, ADAMTS10, and Weill-Marchesani Syndrome

With the identification of *FBN1* mutations in WMS, another *Fbn1* mouse model was developed with deletion of coding exons 9 to 11 of *FBN1* to better understand the physiopathology of WMS. This *Fbn1* ^*WMΔ*^ carries the genomic deletion found in patients and presented a phenotype reminiscent of human WMS. This mouse model did not develop the typical aortic disease found in MFS (Sengle et al., 2012). *Fbn1* ^*WMΔ*^ mice did not present short long bones at birth, but their height was reduced by 3–4 weeks of age in homozygous mice associated with a reduction in lengths of the radius, ulna, and tibia, but also metacarpals, proximal and distal phalanges. These differences were normalized at 5 months of age. Skin analysis suggested thickened and less elastic skin in mutant mice. The staining of collagens showed an increase of collagen deposition, confirmed with the overexpression of the *Col1a1, Col1a2, and Col3a1* genes. Fibrillin-1 fibrils in mutant mice skin were disorganized with an accumulation of large microfibril bundles. Studies on the interactions between mutant FBN1 and partners revealed a decreased interaction with ADAMTSL-2 and -3, thus showing that ADAMTSLs may be involved in the physiopathologic mechanisms of WMS (Sengle et al., 2012). Analysis of the interaction between WMS mutant FBN1 protein and heparan-sulfates showed an almost complete ablated binding, but no differences were observed on cell adhesion (Cain et al., 2012). No aberrant TGF-β signaling activation was found in fibroblasts issues from individuals with WMS (Sengle et al., 2012). The same result was observed in another WMS mouse model, *Adamts10* ^*S236X/S236X*^ (Mularczyk et al., 2018). This WMS mouse model *Adamts10* ^*R236X*^ (using CRISPR Cas9 genome editing and replicating a nonsense mutation from a patient), presented short stature associated with growth plate defects and over-proliferation of chondrocytes within the growth plate (Mularczyk et al., 2018). Eye phenotype showed a decreased ciliary body area, an increase of microfibril bundles and disorganization of FBN1, and overexpression of *Fbn2*. Studies on muscles and skin of the mutant mice showed also decreased activation of the BMP signaling pathway associated with decreased expression of syndecan-4. Adamts10 was shown to be secreted by the cells. Altogether these data suggested that TGF-β signaling may not be involved in the pathomechanism of WMS. Another signaling pathway seems impaired: the p38 mitogen-activated protein kinase.

It has been shown that ADAMTS10 physically interacts with FBN1 (Kutz et al., 2011) to induce microfibril depositions. Mutations in *ADAMTS10* causing WMS lead to mislocalization of the protein, a decrease in the expression, and the N-glycosylation of the protein (Steinkellner et al., 2015), and also in the sparse deposition of FBN1 microfibrils (Kutz et al., 2011). Inactivation of *Adamts10* in mice did not lead to skeletal, cardiovascular, and ocular defects but showed an increased expression of fibrillin-2 (FBN2) (Wang et al., 2019), with thickened skin associated with higher contractility of dermal fibroblasts. The same persistence of FBN2 expression beyond birth was observed in the mouse model, *Adamts10* ^*S236X/S236X*^ *.*

The genetic involvement of ADAMTS10, ADAMTS17, and FBN1 in WMS suggested a potential biological link between these three ECM proteins. Interestingly, both ADAMTS share the same organization but not the same function considering the difference of phenotype between WMS and WMS-like. A major difference resides also in ADAMTS10 resistance to furin cleavage (Kutz et al., 2011). Conversely, ADAMTS17 is correctly processed by furin at the cell surface (Hubmacher et al., 2017). Therefore, ADAMTS10 may have a function as a structural protein and not as an enzyme.

### ADAMTS17, FBN1, and Weill-Marchesani Syndrome-Like

*ADAMTS17* mutations causing WMS seem to decrease the secretion of ADAMTS17 (Evans et al., 2020; Karoulias et al., 2020) or impact the catalytic domain of the protein (Balic et al., 2021), which suggests loss of function mutations leading to an inactive protein. To date, ADAMTS17 has no known substrate except itself *in vitro*. ADAMTS17 usually co-localizes with FBN1 microfibrils, but ADAMTS17 mutant cells failed to form proper FBN1 microfibrils due to intracellular accumulation of FBN1 in lysosomal-like vesicles (Karoulias et al., 2020). These defects lead to elastic fibers abnormalities and a decrease of collagen fibers diameter due to intracellular retention of collagens which exhibit the possible role of ADAMTS17 in collagen biogenesis. An *Adamts17* knock-out mouse model exhibits short stature, brachydactyly, decreased skin elasticity associated with collagen deposition (Oichi et al., 2019). Specific knock-out of *Adamts17* in chondrocytes (using Col2a1-Cre) demonstrate growth plate defects with and increased expression of *Fbn2* and the downregulation of the BMP signaling pathway. These data confirmed the absence of activation of TGFβ signaling pathway in WMS.

To date, all these data can not explain the difference between the WMS and the WMS-like phenotype. Further investigations are needed.

### ADAMTSL2, FBN1, and Geleophysic Dysplasia

Using *in situ* hybridization, ADAMTSL2 is shown to be widely expressed in long bone cartilage (e.g., proliferative and hypertrophic chondrocytes), cardiomyocytes, epidermis, and dermal blood vessels (Le Goff et al., 2008). Although its function is still not defined, as an extracellular component, ADAMTSL2 interacts with FBN2 (Hubmacher et al., 2015), FBN1, and LTBP1 (Le Goff et al., 2011; Le Goff et al., 2008). ADAMTSL2 belongs to the ADAMTSL group without catalytic activity. In skin fibroblasts from GD patients harboring an *ADAMTSL2* gene mutation an excess of TGFβ secretion associated with an increase of the TGFβ signaling pathway has been observed. Using HEK293F cells transfected with ADAMTSL2 plasmids harboring mutations found in GD patients, the secretion of ADAMTSL2 was decreased but no difference in the intracellular level of the protein was observed (Le Goff et al., 2008). Due to previously found intracellular bodies in GD patient cells, the intracellular traffic of ADAMTLS2 was studied. In the skin biopsy of a GD patient harboring an *ADAMTSL2* mutation, lysosomal-like vesicles with a lamellar structure appearance have been found, forming the inclusion bodies (Piccolo et al., 2019). Using site-directed mutagenesis, ADAMTSL2 failed to traffic through the Golgi apparatus leading to decreased extracellular secretion. The low protein rate of ADAMTSL2 in the cellular microenvironment leads to overactivation of the TGFß signaling pathway, which was confirmed by the *Adamtsl2* null mice model (Delhon et al., 2019).

In GD patients carrying *FBN1* mutations, skin fibroblasts showed an increase of active TGFβ in their conditioned medium, associated with an increased TGFβ signaling pathway activation. These dysregulations were correlated with an abnormal organization of microfibrils in GD/AD patient-derived cells (Le Goff et al., 2011). Another study showed that GD and AD FBN1 mutant proteins were secreted and incorporated into microfibrils but failed to form abundant microfibrils. The cellular GD/AD models presented reduced heparan-sulfates binding proprieties. The interaction between heparan sulfate and FBN1 is a major phenomenon in fibrillin assembly. The decrease of binding to heparan sulfate may explain the lower amount of microfibrils in AD/GD ECM (Jensen et al., 2015).

Different *Adamtsl2* null mice models have been generated to decipher the role of ADAMTSL2 during the development (Hubmacher and Apte, 2015; Delhon et al., 2019; Hubmacher et al., 2019). The first model was lethal at birth due to severe bronchial epithelial dysplasia with abnormal glycogen-rich inclusions in the bronchial epithelium. An over activation of TGFβ was observed in E17.5 bronchial epithelium embryos, which was not corrected using TGFβ neutralizing antibodies suggesting that the enhanced activation of the TGFβ signaling pathway may be only a consequence of a disorganized ECM. At the same time, the modulation of microfibril formation by Adamtsl2 has been established. Delhon et al. developed another *Adamtsl2* invalidating mouse model. Its skeletal phenotyping showed short stature of KO mice, with forelimb and hindlimb shortening, impaired digit elongation, ovoid deformation of the vertebrae, and a shortened muzzle and calvaria, reminiscent of the human phenotype (Delhon et al., 2019). Short stature was associated with growth plate defects and alteration of chondrocytes differentiation and proliferation. The absence of Adamtsl2 was associated with an improper Fibrillin-1 and Ltbp1 microfibrillar network, leading to the over-activation of the TGFβ signaling pathway (Delhon et al., 2019). Specific tip-toe walking of GD patients with *ADAMTSL2* mutations led to the establishment of a tendon-specific *Adamtsl*2 deletion mice model (Hubmacher et al., 2019), which also presented short stature associated with a shorter tendon. These studies concluded that the short stature of patients is associated with skeletal development impairments and/or the external tethering by short tendons leading to short long bones.

## Discussion/Conclusion

In the last decade, the discovery of *FBN1* mutations in the acromelic dysplasias group widens the spectrum of fibrillinopathies (Table 1). In fibrillinopathies, disorders present a large spectrum of clinical features from tall to short stature, with or without ectopia lentis. The acromelic dysplasias are considered as the mirror image of MFS, with short stature and brachydactyly. The increase of human genetic data thanks to whole-exome sequencing accelerated the understanding of the group of acromelic dysplasias. The genetic link between ADAMTS and FBN1 suggested a functional link between them.

FBN1 being involved in all these disorders is certainly the central fulcrum in their pathogenic mechanism (Table 2). However, each disease develops specific organ damages that cannot be explained only by the impairment of FBN1-rich microfibrils. Another element of the puzzle is certainly the ADAMTS proteins. To date, specific ADAMTSs have been involved in opposite phenotypes:on the one hand, ADAMTS10, ADAMTS17, and ADAMTSL2 in acromelic dysplasia; on the other hand, ADAMTSL6, a new actor in the Marfanoid phenotype. All four ADAMTS are colocalized with FBN1 microfibrils in ECM of cell cultures or tissues and seem to have a structural rather than an enzyme role (Le Goff et al., 2008; Kutz et al., 2011; Hubmacher and Apte, 2015; Hubmacher et al., 2017). Furthermore, the mutations in each of these ADAMTS lead to distinct phenotypes suggesting that the explanation may reside in distinct tissue-specific functions. Considering the skeletal phenotype of their invalidated mouse models, ADAMTS10, ADAMTS17, and ADAMTSL2 all have a role in endochondral ossification but each mutated protein leads to a distinct syndrome either WMS, WMS-like, or DG (Mularczyk et al., 2018; Delhon et al., 2019; Oichi et al., 2019). Finally and surprisingly, only one ADAMTS, ADAMTSL6, has been implicated in a Marfanoid phenotype until now (Elbitar et al., 2021).

Interestingly, a common clinical characteristic in these mirror image pathologies is ectopia lentis, a lens dislocation that complexes the whole picture. Cases of isolated ectopia lentis have been reported and are related to either FBN1 or ADAMTSL4 gene mutations. But ectopia lentis is also a clinical feature of WMS or MFS with respectively *ADAMTS10/17* or *FBN1* gene mutations (Dagoneau et al., 2004; Morales et al., 2009; Arnaud et al., 2021a). Unlike other organs, in the eye, ADAMTS10, ADAMTS17, FBN1 share a common biological pathway where each partner is essential to the homeostasis of the ciliary zonule. Proteomic analysis showed that ADAMTSL6 is specifically expressed in the human ciliary zonule but to date, no patient carrying a *THSD4* variant has presented ectopia lentis (De Maria et al., 2017) supporting that ADAMTSL4 may compensate for ADAMTSL6 in the eye. Indeed, evolutionarily, ADAMTSL4 and ADAMTSL6 seem to belong to the same clade whereas ADAMTSL2 is apart. It is important to notice that ADAMTSL2, ADAMTSL4, and ADAMTSL6 possess a PLAC domain at C-terminal. To date, no biochemical studies investigate the function of this PLAC domain.

More recent genetic data showed that another molecule may be part of ADAMTS-FBN1 complexes, the LTBP proteins. LTBP2 was the first LTBP protein involved in acromelic dysplasia, WMS-like (Haji-Seyed-Javadi et al., 2012). We also identified *LTBP3* heterozygous mutations in AD and GD patients (McInerney-Leo et al., 2016). The biological cooperation between FBN1 and ADAMTS may be reinforced by LTBP considering the essential role of LTBP in microfibrillar formation (Fujikawa et al., 2017). LTBP3 seems also implicated in TAA considering the recent report of homozygous *LTBP3* variants in TAA patients presenting both a skeletal and a dental phenotype (Guo et al., 2018).

All the genetic and biochemical data suggest strongly that the existence of cooperation between FBN1, ADAMTS, and LTBP. Our recent work on the *Adamtsl2* KO mouse model demonstrated the functional dependency between ADAMTSL2, FBN1, and LTBP1 (Delhon et al., 2019). Moreover, ADAMTS10, ADAMTS17, ADAMTSL2, and ADAMTSL6 colocalize with FBN1 microfibrils in culture cells or mouse models (Le Goff et al., 2008; Tsutsui et al., 2010; Kutz et al., 2011; Hubmacher et al., 2017). ADAMTSL2 binds directly to LTBP1 and FBN1 (Le Goff et al., 2008; Le Goff et al., 2011). ADAMTS10 interacts also with FBN1 in the same region as ADAMTS17 and ADAMTSL6 (Kutz et al., 2011; Hubmacher et al., 2017; Tsutsui et al., 2010). LTBP3 needs the FBN1 microfibrils to be deposited in the ECM (Zilberberg et al., 2012). The exact nature of this cooperation between FBN1, ADAMTs, and LTBP should be more investigated. It remains to establish if these different molecules form a complex and how these complexes can explain the distinct phenotypes.

One incomprehensible point resides in the potential involvement of the TGFβ signaling pathway in both acromelic dysplasias and MFS. In AD/GD patient-derived fibroblasts, an increase of the TGFβ signaling pathway was noticed (Le Goff et al., 2008; Le Goff et al., 2011). The two different *Adamtsl2* KO mouse models showed also an elevated TGFβ signaling activation in different tissues (lung or growth plate) (Hubmacher et al., 2015; Delhon et al., 2019). But the use of TGFβ neutralizing antibodies failed to rescue the broncho-insufficiency (Hubmacher et al., 2015). In AD and GD, the TGFβ signaling may be a consequence of the dysregulation of the ECM in the growth plate or the lung. No specific activation of TGFβ signaling was observed in WMS (Sengle et al., 2012). Recent data on a decrease of BMP signaling associated with increased p38MAPK signaling in WMS complicates the whole picture of the pathomechanism of acromelic dysplasias (Mularczyk et al., 2018). The TGFβ seems not to be the essential pathogenic driver in WMS. More importantly, the existence of *SMAD4* mutations in Myhre syndrome, another acromelic dysplasia corroborates the involvement of TGFβ and/or BMP signaling, SMAD4 being a central mediator of these two pathways (Le Goff et al., 2012). In these patient-derived fibroblasts, abnormal ECM was observed (Piccolo et al., 2014). These results reinforce the possibility that the involvement of TGFβ/BMP is only a response to the anomalies of the ECM. In patients with *THSD4* mutations, an impairment of the TGFβ signaling pathway has been established (El Bitar et al., 2021) confirming the role of TGFβ in diseases belonging to the Marfanoid spectrum.

To conclude, the genetic findings constitute the main source of information on the potential role of these ADAMTS proteins. New genetic data will permit a better understanding of the pathomechanism of these opposite phenotypes. Indeed, we suppose that all the genes of these pathologies have not yet been identified. A common point in all these pathologies involving ADAMTS proteins is the default in the microfibrillar network. We propose that these disorders could be grouped in a group named the microfibrillinopathies. Ongoing studies will give us decipher the existence and exact role of these complexes formed by the ADAMTSs, FBN1, and LTBP in these microfibrillopathies.
